# Role of RNA N6-Methyladenosine Modification in Male Infertility and Genital System Tumors

**DOI:** 10.3389/fcell.2021.676364

**Published:** 2021-05-19

**Authors:** Shuai Liu, Yongfeng Lao, Yanan Wang, Rongxin Li, Xuefeng Fang, Yunchang Wang, Xiaolong Gao, Zhilong Dong

**Affiliations:** ^1^Department of Urology, Lanzhou University Second Hospital, Lanzhou, China; ^2^Gansu Nephro-Urological Clinical Center, Institute of Urology, Department of Urology, Key Laboratory of Urological Disease of Gansu Province, Lanzhou University Second Hospital, Lanzhou, China; ^3^Department of Urology, People’s Hospital of Jinchang, Jinchang, China; ^4^Second Clinical Medical College, Lanzhou University, Lanzhou, China; ^5^Xiangya Hospital, Central South University, Changsha, China

**Keywords:** N6-methyladenosine, RNA methylation, spermatogenesis, male fertility, genital system tumors

## Abstract

Epigenetic alterations, particularly RNA methylation, play a crucial role in many types of disease development and progression. Among them, N6-methyladenosine (m6A) is the most common epigenetic RNA modification, and its important roles are not only related to the occurrence, progression, and aggressiveness of tumors but also affect the progression of many non-tumor diseases. The biological effects of RNA m6A modification are dynamically and reversibly regulated by methyltransferases (writers), demethylases (erasers), and m6A binding proteins (readers). This review summarized the current finding of the RNA m6A modification regulators in male infertility and genital system tumors and discussed the role and potential clinical application of the RNA m6A modification in spermatogenesis and male genital system tumors.

## Introduction

Over the last decades, with the rapid development of gene detection technology, epigenetic modification of diseases has become a focus of clinical research. Epigenetics is a branch of genetics that studies heritable and reversible phenotypes of changes in gene expression without alterations in nuclear DNA sequences (Mohammad et al., 2019). Epigenetic processes, including DNA methylation, histone modifications, chromatin rearrangement, and RNA modifications, play a crucial role in the regulation of many physiological and pathological processes, such as embryonic development (Mendel et al., 2018), nervous system development (Li M. et al., 2018), and tumorigenesis (Chen S. et al., 2020; Sun Y. et al., 2020). Therefore, the regulation of these epigenetic processes may be a potential therapeutic intervention. m6A is one of the most common types of eukaryotes in RNA modification (Dominissini et al., 2012; Meyer et al., 2012). Since the pioneering research in the 1970s (Desrosiers et al., 1974; Perry et al., 1975), with the identification of more m6A-related enzymes, the important biological functions played by m6A modification have been gradually revealed around about half a century later.

Since the pioneering research in the 1970s (Desrosiers et al., 1974; Perry et al., 1975), with the identification of more M6A-related enzymes, the important biological functions played by m6A modification have been gradually revealed around about half a century later. With the first m6A demethylase—FTO was found in 2011, more m6A regulators have been found in the past decade which could be divided into three kinds of regulator proteins: methyltransferases (“writer”), such as methyltransferase-like protein 3 (METTL3), methyltransferase-like protein 14 (METTL14), Wilms’ tumor 1-associating protein (WATP) (Liu et al., 2014; Ping et al., 2014; Li Z. et al., 2018), Vir-like m6A methyltransferase-associated (VIRMA; also known as KIAA1429) (Yue et al., 2018); demethylases (“erasers”), such as obesity-associated protein (FTO) and AlkB family homolog 5 (ALKBH5) (Jia et al., 2011; Zheng et al., 2013); and m6A binding proteins, such as the YTH domain family proteins (YTHDFs) and YTH domain-containing protein 1-2 (YTHDC1-2) (Dominissini et al., 2012; Wang et al., 2014, 2015), The insulin-like growth factor 2 mRNA binding proteins (IGF2BPs) (Huang et al., 2018), heterogeneous nuclear ribonucleoprotein A2B1 (HNRNPA2B1) (Alarcón et al., 2015), and eukaryotic translation initiation factor 3 (eIF3) (Meyer et al., 2015).

With changes in environmental pollution and living habits, the incidence and prevalence of male infertility and andrology tumors increase steadily worldwide and have become a prevalent worldwide problem in recent decades. About 2-15% of couples worldwide are reported to suffer from infertility, of which nearly half are caused by male reproduction (Ostermeier et al., 2002; Ji et al., 2012), a large proportion of male infertility was related to defective spermatogeneses, such as abnormal sperm count, morphology, and function (Guzick et al., 2001). Female infertility can usually be induced by hormone therapy to stimulate the production of oocytes, while male infertility is more difficult to treat due to various reasons for sperm abnormalities (Tournaye et al., 2017). Surgery is the primary treatment option for men with obstructive azoospermia and part of non-obstructive infertility such as varicocele and undescended testicles at birth (Dubin and Amelar, 1971; Verza and Esteves, 2008). Recently, intracytoplasmic sperm injection (ICSI) is a relatively novel approach to assisted reproduction that has brought revolutionary changes in clinical therapy for infertility (Kyono et al., 2001). But there are still a large proportion of patients who cannot meet the wish of fatherhood. Therefore, scientists gradually realize that it is very important to study the causes of male infertility.

Another disease that severely impairs male health and quality of life is genital system tumors. Prostate cancer (PCa) is the most common malignant tumor of the male genital system and its pathogenesis involves multiple factors, which mainly affects elderly men (Siegel et al., 2021). Although the mortality rate of prostate cancer is not high (Tsubokura et al., 2018), due to the molecular mechanisms underlying the development and progression of prostate cancer remain unclear as well as its high incidence, it is worth studying and its treatment is of great significance for the longevity of the elderly. Additionally, the most common reproductive system tumors in men aged 15-35 years old are testicular germ cell tumors with increasing incidence in recent years (Ghazarian et al., 2017). Most testicular germ cell tumors (GCTs) are completely curable with cisplatin-based chemotherapy. However, a small proportion of patients who cannot be cured with cisplatin chemotherapy should be the focus of our research. Therefore, greater insights into the mechanisms regulating spermatogenesis and male genital system tumors will help us found novel molecular targets to develop more effective treatment strategies for the disease. In this review, we have provided an overview of the underlying m6A methylation and the mechanisms of development of spermatogenesis and andrology tumors. We further highlight the potential use of m6A methylation modification as a biomarker for andrology diseases risk diagnosis, therapeutic applications, and prognosis.

## RNA M6A Modification

**FIGURE 1:**
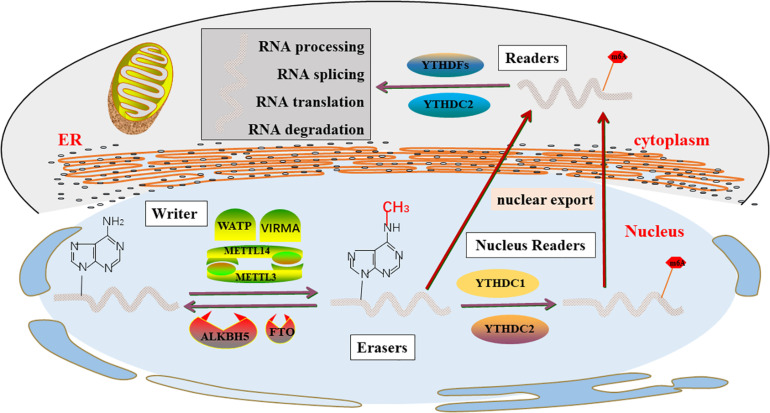
m6A enzyme molecular mechanisms in detail. The “writers”, “Erasers”, and “Readers” rely on a variety of related factors to cause m6A modifications through installation, removal and combination and involved in RNA metabolism, including processing, degradation, splicing, and translation.

The important biological functions played by mRNA m6A dynamic and reversible modification are mainly catalyzed by m6A related enzymes. A large number of studies have shown that the m6A methylation of mRNA modification was catalyzed by three elements (Figure 1).

### Methyltransferases (“Writers”)

The methylation process is first controlled by a family of enzymes called methyltransferases, also known as a writer, which are proteins that induce specific RNA methylation, which catalyzes the formation of m6A levels. In 1997, METTL3 was first shown to be m6A methylation transferase, and the expression of METTL3 can direct effects the total methylation level of m6A, which has effects on mRNA stability, leading to dysregulated cellular functions (Bokar et al., 1997). Several studies (Śledź and Jinek, 2016; Wang P. et al., 2016; Wang X. et al., 2016) suggest that mRNAs methyltransferases are primarily formed by the METTL3-METTL14 heterodimer, of which METTL3 is a catalytic active subunit and METTL14 plays a key structural role in substrate recognition, maintaining complex integrity, and substrate binding.

As rapid and sensitive detection technology advances, more methyltransferases were found. METTL5 and METTL16 is a methyltransferase that targets pre-mRNAs and various non-coding RNAs, such as rRNA, snRNA (Pendleton et al., 2017; Warda et al., 2017; Wojtas et al., 2017; van Tran et al., 2019; Ignatova et al., 2020). WATP is another m6A “writer” complex, which stabilizes the core complex and targets the METTL3 and METTL14 complexes to their substrates (Ping et al., 2014). VIRMA prioritizes gene methylation modifications near the 3’-UTR and stop codon regions and recruits the m6A complex to specific RNA sites (Yue et al., 2018). ZC3H13 anchors WTAP to the nucleus to promote m6A methylation and regulation (Wen et al., 2018). Recently, another CCHC-containing zinc finger protein, ZCCHC4, has been identified as a novel methyltransferase, which is involved in the modification of 28S rRNA and mediates the subunit distribution and global translation of rRNA ribosomes (Ma et al., 2019; Ren W. et al., 2019; Pinto et al., 2020).

### Demethylases (“Erasers”)

RNA m6A modification was mainly removed by demethylases FTO and ALKBH5, which were an essential enzyme in m6A modification so that maintained m6A modification in a dynamic balance, and dynamically regulated developmental and disease processes (Jia et al., 2011; Zhang X. et al., 2020). FTO, originally known as an obesity-susceptibility gene, is strongly associated with obesity risk (Dina et al., 2007; Frayling et al., 2007; Scuteri et al., 2007). A subsequent study (Jia et al., 2011) demonstrated that FTO is the first m6A demethylase of eukaryotic mRNA and the role of FTO in adipogenesis and tumorigenesis is related to its m6A demethylase activity. The majority of studies (Söderberg et al., 2009; Castillo et al., 2012; Iles et al., 2013; Li et al., 2017) have shown a strong association between FTO and an increased risk of various types of cancer, including breast cancer, prostate cancer, kidney cancer, endometrial cancer, pancreatic cancers, lymphoma, and leukemia. These studies reveal the roles and underlying molecular mechanisms of FTO in cancer pathogenesis. Therefore, the development of selective and effective inhibitors targeting FTO will have the potential to treat cancer, particularly in combination with other therapies to treat cancers that are resistant to currently available therapies. ALKBH5 is another m6A eraser that is localized in the nucleus, which is most highly expressed in the testicles. Therefore, it may be necessary for mouse spermatogenesis and fertility (Zheng et al., 2013; Tang et al., 2018). Available pieces of evidence indicate that ALKBH5 mainly inhibits the development of a variety of cancers.

Taken together, as m6A demethylases, FTO and ALKBH5 have opposite effects in some tumors, and the specific reasons are worth further study. In addition, recent studies have shown that ALKB family homolog 3 (ALKBH3) may be a novel demethylase modified by m6A, and modified mammalian tRNA demethylation promotes protein synthesis in cancer cells (Ueda et al., 2017).

### m6A Binding Proteins (“Readers”)

The effect of m6A modification is primarily dependent on downstream RNA-binding proteins, known as m6A “readers”, which prioritize the recognition of m6A modified RNA and combine m6A methylation with RNA processing and biological functions (Li F. et al., 2014; Zhu et al., 2014). Recent studies have shown that m6A modification regulates most RNA processing steps, including mRNA translation (Tanabe et al., 2016; Shi et al., 2017; Mao et al., 2019), mRNA splicing (Hartmann et al., 1999; Xiao et al., 2016; Kasowitz et al., 2018; Luxton et al., 2019), stability (Du et al., 2016), and transport (Roundtree et al., 2017). In the known m6A readers, most proteins contain a YTH domain that specifically recognizes m6A and A (Stoilov et al., 2002), including YTHDC1 in the nucleus (Hartmann et al., 1999), YTHDFs family in the cytoplasm (Wang et al., 2014), and YTHDC2 in the nucleus and cytoplasm (Wojtas et al., 2017).

YTHDC1 increases Akt phosphorylation by promoting PTEN mRNA degradation, which promotes neuronal survival, especially after ischemia (Zhang Z. et al., 2020). The lack of YTHDC1 in oocytes impedes its development at the primary follicular stage, additionally, result in a large number of selective splicing defects in oocytes (Kasowitz et al., 2018). Specifically, YTHDC1 is required for female oocyte growth and maturation. Chen H. et al. (2020) conducted a seven-center case-control study in Chinese children concluded that YTHDC1 gene polymorphism may have a cumulative effect on the susceptibility of hepatoblastoma oncogene. This study suggests that YTHDC1 might be a potential biomarker and therapeutic target for certain diseases, not only the biological profile of the malignant disease.

According to previous researches, YTHDC2 may play an important role in a variety of diseases genesis, and development. More recent studies suggest that YTHDC2 is a potential candidate gene for pancreatic cancer susceptibility (Fanale et al., 2014) and is associated with immune infiltration in head and neck squamous cell carcinoma (Li Y. et al., 2020). In lung adenocarcinoma, it suppresses the tumorigenesis and development by inhibiting SLC7A11-dependent antioxidant function (Ma et al., 2021). In non-small cell lung cancer, downregulation of m6A reader YTHDC2 promotes tumor progression and predicts poor prognosis (Sun S. et al., 2020). YTHDC2 may promote the metastasis of colon cancer by promoting the translation of HIF-1α, and YTHDC2 may be a diagnostic marker and target gene for the treatment of colon cancer (Tanabe et al., 2016). Zeng et al. (2020) suggest that mRNA m6A modification and YTHDC2 expression are crucial to meiotic initiation and progression in female germ cells. And YTHDC2 also regulates the transition from proliferation to differentiation of germline (Bailey et al., 2017).

Another recognized YTH domain-containing protein member is YTHDF1-3, which all have a conserved m6A-binding domain. In 2020, Liu J. et al. (2020) shown that autophagy YTHDF2/3 is required for pluripotent stem cells reprogramming. Shi et al. (2019) found that high expression of YTHDF1 is associated with better hypoxic adaptation suppression of non-small cell lung cancer (NSCLC), however when its depletion causes cancer cells to develop resistance to cisplatin (DDP) therapy via the KEAP1-NRF2-AKR1C1 axis, especially the accumulation of reactive oxygen species (ROS) induced by cisplatin treatment. Also, By stabilizing MAP2K4 and MAP4K4mRNA transcription, YTHDF2 activates MAPK and NF-κB signaling pathways promotes the expression of pro-inflammatory cytokines, and aggravates the inflammatory response (Yu et al., 2019).

In eukaryotic cells, in addition to the well-characterized YTH protein, there is also a unique m6A “code reader” protein. These readers recognize and directly and specifically bind to the m6A site, which plays an important role in RNA metabolism. The IGF2BP family is newly reported m6A readers, which are consisted of three members of IGF2BP1-3 (Huang et al., 2018). These proteins are responsible for the stability of targeted mRNAs and are associated with thousands of targets, such as MDR1, MYC, and KRAS (Bell et al., 2013). In short, IGF2BPS recognizes mRNAs modified by m6A and promotes cancer progression by recruiting RNA stabilizers, thus maintaining their stability (Huang et al., 2018; Li et al., 2019; Hanniford et al., 2020).

The primary role of eIF3 is to facilitate translation. Protein translation typically begins with the recruitment of the 43S ribosomal complex to the 5′ cap of mRNAs by a cap-binding complex. However, m6A directly binds eukaryotic initiation factor 3 (eIF3) and recruits the 43S complex to initiate translation in the absence of the cap-binding factor eIF4E (Meyer et al., 2015). Recently, Lee et al. (2016) revealed that eIF3d (a subunit of the eIF3 complex) is an mRNA cap-binding protein required for specific translation initiation. DAP5, an eIF4GI homolog that lacks eIF4E binding, which promotes cap-dependent translation by combining directly with eIF3d (de la Parra et al., 2018). (Alarcón et al., 2015) found that the RNA-binding protein hnRNPA2B1 binds to the m6A RNA, whose biochemical footprint matches the m6A common motif, and proposed that hnRNPA2B1 is A nuclear reader of m6A markers, and modulated the effect of this marker on the processing and selective splicing of primary microRNAs to A certain extent.

## Role of RNA M6A Modification in Male Fertility

**FIGURE 2:**
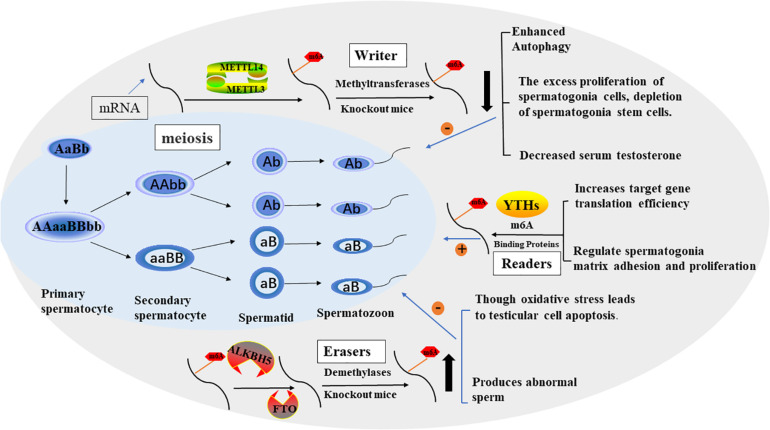
The process of spermatogenesis and the potential role of m6A in spermatogenesis. The effect of M6A on male fertility is reflected in the regulation of spermatogenesis. In general, m6A modification promotes sperm production through various factors.

Infertility is one of the common health problems in modern society, with about 50 percent of problems being caused by male factors (Drobnis and Johnson, 2015; Whitfield et al., 2015). Sperm defects are the primary cause of male infertility, including sperm concentration, sperm motility, and morphology (Huynh et al., 2002). In recent years, A growing number of studies have shown that modification of germ cell RNA m6A can lead to defects in the process of spermatogenesis, leading to male infertility, and which can not be cured by assisted reproductive technology (Table 1 and Figure 2). So we will discuss the effects of different enzymes modified by m6A on spermatogenesis, which provides a novel insight for improving the therapeutic effect of this kind of patients, which may be beneficial to the further clinical application of this kind of patients in male infertility treatment.

**TABLE 1:** Roles of m6A proteins and biological mechanisms exerted in spermatogenesis.

**Type**	**Regulator**	**role in spermatogenesis**	**Functional classification**	**References**
Writers	METTL3	Blocked the initiation of meiosis.	Loss of m6A severely inhibited spermatogonia differentiation.	Xu et al., 2017
	METTL3/14	Promoting SSC/progenitor cell proliferation and differentiation.	Loss of m6A leads to dysregulated translation of SSC/progenitor cell and causing SSC depletion.	Lin et al., 2017
	METTL14\ALKBH5	Regulates testosterone synthesis through modulating autophagy in Leydig cells.	Reduced mRNA methylation levels of m6A and enhanced autophagy in LCs.	Chen Y. et al., 2020
	METTL3	Regulating the expression of genes critical for sex hormone synthesis and gonadotropin signaling.	Loss of METTL3 leads to failed gamete maturation and significantly reduced fertility in zebrafish.	Xia et al., 2018
Erasers	ALKBH5	Impaired fertility resulting from apoptosis that affects meiosis metaphase-stage spermatocytes.	ALKBH5 deficiency leads to compromised spermatogenesis in mice.	Zheng et al., 2013
	ALKBH5	Controls splicing and stability of long 3’-UTR mRNAs in male germ cells.	Dysregulation of many genes could contribute to meiotic defects.	Tang et al., 2018
	FTO\YTHDC2	Inhibition of demethylase FTO contributes to MEHP -induced Leydig cell injury.	Leading to aggravated oxidative stress and testicular injury.	Zhao et al., 2020, 2021
	FTO	A associated with reduced semen quality.	Single nucleotide variants could cause a shift in the transcription of the gene.	Landfors et al., 2016
Readers	YTHDC2	Regulates a meiotic in the mammalian germline.	Loss of YTHDC2 results in upregulation of several genes that are normally expressed in the mitotic spermatogonia and downregulation of meiotic genes.	Jain et al., 2018; Wojtas et al., 2017
	YTHDC2	Regulates the transition from proliferation to differentiation in the germline.	The proper progression of germ cells through meiosis is licensed by YTHDC2 through post-transcriptional regulation.	Bailey et al., 2017
	YTHDC2	Regulates mammalian spermatogenesis.	Lacking YTHDC2 are infertile and do not contain germ cells able to develop beyond the zygotene stage of meiotic prophase I.	Hsu et al., 2017
	YTHDC2	Promotes spermatogonia adhesion.	Depletion of YTHDC2 mainly downregulated the expression of MMPs, thus affecting cell adhesion and proliferation.	Huang et al., 2020
				

Mammalian spermatogenesis is a highly specialized differentiation process involving multiple regulatory mechanisms, and m6A can influence pre-mRNA splicing, mRNA output, turnover, and translation, which are controlled in the male germline to ensure coordinated gene expression. In 2019, Yang et al. (2016) collected semen samples and tested the content of m6A in sperm ribonucleic acid using liquid mass spectrometry and the expression of m6A-modified proteins using real-time polymerase chain reaction. The results showed that increased M6a content was a risk factor for asthenozoospermia and affected sperm motility. Methyltransferases, especially METTL3, play a key role in increasing the amount of m6A in sperm RNA. Xu et al. (2017) found that METTL3 regulates spermatogonia differentiation and controls meiosis initiation in germ cells. Lin et al. (2017) proposed the m6A mRNA methylomes of mouse spermatogenic cells from the five developmental stages: undifferentiated spermatogonia, A1 type spermatogonia, pre-embryonic spermatogonia, pachytene/diploid spermatogonia, and round spermatogonia. The study highlights the key role of m6A gene modification in germline development and the potential to ensure coordinated translation at different stages of spermatogenesis. YTHDC2 improves the translation efficiency of target genes, affects the testicular volume of male mice, and enables the development of germ cells in mice, which plays a key role in spermatogenesis (Hsu et al., 2017). The sterile mutant “ketu”, caused by YTHDC2 missense mutation, makes the mutant germ cells enter meiosis, but prematurely enter abnormal metaphase and apoptosis. And defective genes that lead from spermatogonia to meiosis (Jain et al., 2018). A recent study (Tang et al., 2020) shows that in mouse spermatogenesis, the start and stop codon of linear ribonucleic acid is usually located around the m6A enriched sites, causing late pachytene spermatocytes to develop into round cells, which then extend into spermatoblast cells.

### Methyltransferases (“Writers”) in Spermatogenesis

In 2017, Xu et al. (2017) shown that loss of METTL3 in germ cells severely inhibits spermatogonia differentiation and blocks the onset of meiosis by cultivating germ cell-specific METTL3 knockout mice. Therefore, they demonstrated that METTL3 is necessary for male fertility and spermatogenesis. Consistent with this, Lin et al. (2017) found that the m6A RNA methyltransferase METTL3 and METTL14 proteins colocalize to the nucleus of male germ cells. m6A deficiency caused by germ cell-specific inactivation of METTL3 and METTL14 can lead to excessive proliferation of spermatogonial cells, which would, in turn, result in depletion of spermatogonial stem cells. Immediately after studies proved (Chen Y. et al., 2020) there was a negative correlation between m6A methyltransferase METTL14 and autophagy in testicular stromal cells. The regulation of autophagy in testicular stromal cells can affect the synthesis of testosterone. So, it provides insights into therapeutic strategies for azoospermia and oligozoospermia in patients with reduced serum testosterone. Xia et al. (2018) found defects in sperm maturation and sperm motility are significantly reduced in m6A methyltransferase METTL3 mutant zebrafish. Meanwhile, they pointed that m6A METTL3 is playing important role in the expression of genes critical for sex hormone synthesis and gonadotropin signaling.

### Demethylases (“Erasers”) in Spermatogenesis

Zheng et al. (2013) were detected the ALKBH5 gene’s highest expression levels in testes. m6A gene was increased in male mice with ALKBH5-targeted deletion, and the number of sperm released and incised caudal epididymis were significantly reduced, sperm morphology was abnormal and motility was greatly reduced. The results showed that fertility was impaired due to the abnormal apoptosis and production of a small number of abnormal spermatozoa during meiosis. Tang et al. (2018) came to a similar conclusion, male mice with ALKBH5-targeted deletion testes were approximately half of the size of wild-type controls, the apoptotic germ cells in testis increased, the meiosis process was delayed, and the active germ cells decreased. Meanwhile, they found that m6A tends to label the 3 ‘-UTR of longer mRNAs destined to be degraded during spermatogenesis to keep the stability of long 3’-UTR mRNAs and prevent abnormal splicing to produce shorter transcripts that rapidly degrade.

Landfors et al. (2016) found that FTO genetic variant to be associated with decreased semen quality. In Zhao et al. (2020) conduct a series of studies (Zhao et al., 2020, 2021) demonstrated that Di-(2-Ethylhexyl) phthalate increases m6A RNA modification, deteriorates testicular histology, reduces testosterone concentration, down-regulates spermatogenesis inducer expression, enhances oxidative stress, and increases testicular cell apoptosis by altering the expression of two important RNA methylation regulatory genes, FTO and YTHDC2. These findings link oxidative stress imbalance to the epigenetic effects of DEHP toxicity and provide insights into the testicular toxicity of DEHP from a new perspective of m6A modification.

### m6A Binding Proteins (“Readers”) in Spermatogenesis

Several previous studies (Bailey et al., 2017; Hsu et al., 2017; Wojtas et al., 2017) have shown that YTHDC2 improves the translation efficiency of target genes, Unlike other commonly expressed YTH proteins, YTHDC2 is enriched in the testis. The YTHDC2 knockout mice showed defects in spermatogenesis but no other significant developmental defects. Male mice with YTHDC2 knockout were infertile and the germ cells don’t develop to the zygotic stage. The sterile mutant “ketu” is a missense mutant of the YTHDC2 gene, which causes defects in the transition of germ cells to the meiotic RNA expression program (Jain et al., 2018). Thus, the regulation of M6A transcription by YTHDC2 is the key to the success of meiosis in the mammalian germline. Recent research (Huang et al., 2020) demonstrated that YTHDF2 also regulates the expression of MMPs through the m6A/mRNA degradation pathway to regulate spermatogonia cell-matrix adhesion and proliferation.

Therefore, it can be speculated that m6A plays a major regulatory role in sperm production and development, and provides a new direction for the treatment of male infertility, but further exploration is needed.

## Role of RNA M6A Modifications in Male Genital System Tumors

**FIGURE 3:**
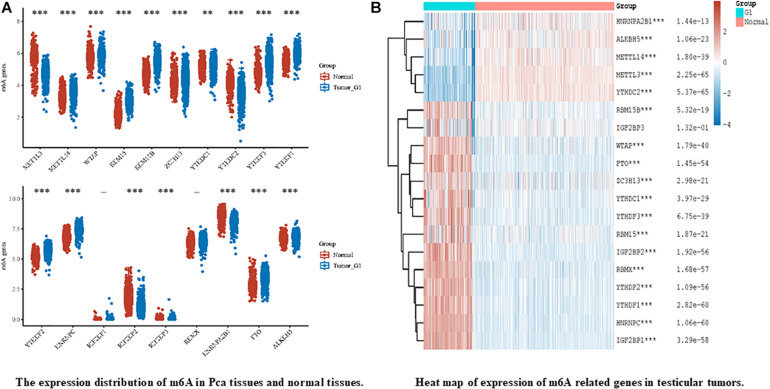
Expression release map and expression heat map of m6A related genes in male genital system tumors, in which different colors represent expression trends of different groups and m6A in different samples. **(A)** represents the expression of m6A in prostate cancer (Pca) and normal prostate tissue, The blue bar chart represents Pca and the gray bar chart represents normal prostate tissueand. Asterisks represents the correlation. **(B)** on behalf of the heatmap of m6A-related genes in testicular germ cell tumors (TGCTs). The left part of figure represents TGCTs, and right represents normal testicular germ cell. Heat map color key: red to blue represents decrease of p value. Where “-” represents no significant difference, *p < 0.05, **p < 0.01, ***p < 0.001. P value less than 0.05 indicates statistically significant results. The significance of the two groups of samples passed the Wilcox test. All data from TCGA and GTEx datasets. All the above analysis methods and R package were implemented by R foundation for statistical computing (2020) version 4.0.3 and software packages ggplot2 and pheatmap.

**FIGURE 4:**
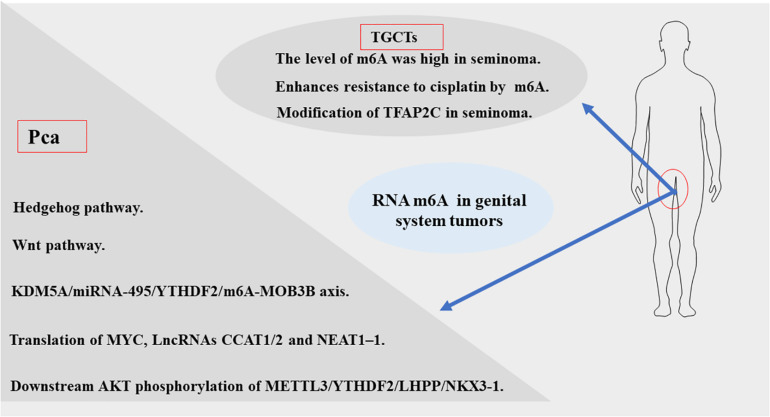
The momentous biological pathways of m6A exertedin genital system tumors.

In recent years, breakthroughs have been made in the diagnosis and treatment of male genital system tumors (Nilsson et al., 2009; Chovanec et al., 2018; Lobo et al., 2019b). However, morbidity and mortality rates remain high. This indicates an urgent need to understand the mechanisms of metastasis and drug resistance of tumors. Recent evidence suggests that RNA m6A methylation is closely related to the development and progression of genital system tumors, including carcinogenesis, proliferation, metastasis, and tumor suppression. According to the existing literature, m6A modifications in genital system tumors are mainly concentrated in prostate cancer and testicular germ cell tumors. Therefore, we firstly analyzed the expression of the m6A regulator in prostate cancer and testicular tumors using databases such as The Cancer Genome Atlas (TCGA) dataset^1^ (Figure 3). The results also showed that m6A related genes were closely related to male genital system tumors. Next, we briefly review the mechanism of m6A methylation in prostate cancer, testicular germ cell tumors, and seminoma for future treatment (Table 2 and Figure 4).

**TABLE 2:** Roles of m6A proteins and biological mechanisms exerted in genital system tumor.

**Cancer**	**Regulator**	**Role in cancer**	**Mechanism**	**Functional classification**	**References**
PCa	METTL3	Oncogene	By regulating hedgehog pathway.	Promotes the growth and motility of prostate cancer cells.	Cai et al., 2019
	METTL3	Oncogene	Influences the activity of the *Wnt* pathway through m6A methylation on LEF1 mRNA.	Promoting the progression of PCa.	Ma et al., 2020
	METTL3	Oncogene	Through mediating MYC methylation.	Promotes the development and progression of PCa.	Yuan et al., 2020
	METTL3	Oncogene	Stabilizing integrin β1 mRNA via an m6A-HuR-dependent mechanism.	Affects the binding of ITGB1 to Collagen I and tumor cell motility to promote the bone metastasis of PCa.	Li E. et al., 2020
	METTL3	Oncogene	NEAT1–1 promoted the binding between CYCLINL1 and CDK19 and the Pol II ser2 phosphorylation.	Associated with prostate cancer aggressiveness.	Wen et al., 2020
	FTO	Oncogene	/	The decrease in FTO increases m6A levels to inhibit PCa cell invasion	Zhu et al., 2021
	VIRMA	Oncogene	Regulate the expression of lncRNA CCAT1 and CCAT2.	Augmenting PCa cell proliferation, migration and invasion *in vitro*, and	Barros-Silva et al., 2020
	YTHDF2	Oncogene	Activation of the KDM5A/miRNA-495/YTHDF2/m6A-MOB3B axis.	Promoting tumor growth.	Du et al., 2020
TGCTs	METTL3/YTHDF2	Oncogene	Through the downstream AKT phosphorylation of METTL3/YTHDF2/LHPP/NKX 3-1.	Induce tumor proliferation and migration.	Li J. et al., 2020
	METTL3	Oncogene	TFAP2C promotes TCam-2 cell survival and confers resistance to CDDP in seminoma.	Potentiates resistance to cisplatin.	Wei et al., 2020
	m6A	Oncogene	m6A levels in RNA increase upon differentiation of TGCT cell lines.	m6A can be detected in RNA of TGCT in different tissues and germ cells at different developmental stages.	Nettersheim et al., 2019
	VIRMA/YTHDF3	Oncogene	over-expression in seminoma.	VIRM6A and YTHDF3 transcript levels accurately discriminate SEs and NSTs.	Lobo et al., 2019a

*PCa: prostate cancer; TGCTs: testicular germ cell tumors; SEs: seminoma; NSTs: non-seminomatous tumor; TFAP2C: transcription factor-activating enhancer-binding protein 2C; TCam-2: seminoma cell line; CDDP: cisplatin (cis-diamminedichloroplatinum II).*

### Prostate Cancer

Globally, about 1.3 million new cases of prostate cancer and 359,000 associated deaths worldwide are reported in 2018, ranking as the second most common cancer and the fifth most leading cause of cancer death in men (Bray et al., 2018). In 2021, in America estimated about 248,530 new prostate cancer cases account for 26% of all incident cases in men, and the second most leading cause of cancer death in the United States (Siegel et al., 2021). Although there are many treatments available, such as androgen-deprivation therapy, a small number of patients still develops resistance (Castration-Resistant Prostate Cancer, CRPC), and progress to lethal metastatic disease (mCRPC). Therefore, identifying novel molecular targets, and understanding the mechanisms driving PCa is of critical importance to treatment. Since 2019, several groups have explored the mechanisms of m6A in prostate cancer.

Cai et al. suggested that upregulation of m6A methyltransferase METTL3 promotes the growth and movement of prostate cancer cells by the Hedgehog pathway for the first time in 2019 (Cai et al., 2019). In 2020, several studies have shown that m6A writer MELLT3 and VIRMA serve an oncogenic role to promotes the development and progression of PCa by Wnt pathway (Ma et al., 2020) and promoting the translation of MYC (Yuan et al., 2020), lncRNAs CCAT1/2 (Barros-Silva et al., 2020). Other studies have proposed that MELLT3 regulates the expression of Integrin β1 (ITGB1) and a high m6A level of LncRNA NEAT1–1 promotes the bone and lung metastasis of PCa (Li E. et al., 2020; Wen et al., 2020). Du and Li et al. showed that YTHDF2 performed Oncogene functions in PCa through the KDM5A/miRNA-495/YTHDF2/m6A-MOB3B axis and the downstream AKT phosphorylation of METTL3/YTHDF2/LHPP/NKX 3-1, respectively (Du et al., 2020; Li J. et al., 2020). According to a new study in 2021 (Zhu et al., 2021), the m6A demethylase FTO inhibits the invasion and migration of prostate cancer cells by regulating and reducing the total m6A level. Wu et al. found the abnormal expression of m6A modification-related enzymes in PCa leads to the elevated m6A level, which is related to Gleason classification (Wu et al., 2021). Thus, based on the current study, we can be concluded that m6A levels may contribute to the development and progression of PCa. But the specific mechanism still needs to be further explored.

### Testicular Germ Cell Tumors

Testicular tumors are relatively rare, accounting for only 1-2% of all tumors in men. The testicular germ cell tumor (TGCTs) accounts for 95% of testicular carcinoma, which is divided into seminoma and non-seminoma histologically (Walsh et al., 2006), while seminoma represents more than 55% of germ cell tumors (GCTs) (Chia et al., 2010).

Nettersheim et al.’s studies (Nettersheim et al., 2019) have shown that RNA levels of m6A were increased during differentiation of GCT cell lines. Lobo et al. (Lobo et al., 2019a) found that the abundance of m6A and the expression of VIRMA/YTHDF3 differed among TGCT subtypes, and the level of m6A was high in seminoma (SE), which accurately distinguished SEs and non-seminomatous tumor (NSTs), forming a new candidate biomarker for patient management. Testicular germ cell tumors are significantly sensitive to anticancer drug cisplatin (Motzer et al., 1991; Einhorn, 1993), which contributes to an overall good prognosis. However, some patients have developed resistance to platinum-based treatments, the emergence of cisplatin (CDDP) resistance is the main cause of treatment failure and death in patients with testicular germ cell tumors (TGCT), but its biologic background is poorly understood (Motzer et al., 1991; Bagrodia et al., 2016; Bakardjieva-Mihaylova et al., 2019). Recently, Wei et al. (2020) found that METTL3 enhances resistance to cisplatin by m6A modification of TFAP2C in seminoma. According to the above evidence, m6A modification plays an important carcinogenic role in the development and progression of TGCT. Therefore, the m6A related genes can be used as a prognostic indicator of early TGCT and may be a potential therapeutic target to prevent the progression of TGCT.

## The Potential Application of RNA m6A in Clinical

Although there are rarely studies on the use of m6A in andrology, for the treatment of other diseases which points us in the direction of research in the future. The treatment of PD-1 has achieved great success in the clinic. However, only a small proportion of cancer patients benefit from PD-1 blocking therapy, and overcoming resistance to PD-1 blocking has become a priority. METTL3/14 can regulate immune responses to PD-1-treated tumors (Wang et al., 2020). Yang et al. (2019) shown that knocking out FTO made melanoma cells hypersensitive to interferon γ (IFN-γ) and PD-1 therapy in melanoma. Li N. et al. (2020) found that in melanoma, colorectal, loss of the m6A demethylase ALKBH5 makes tumors sensitive to cancer immunotherapy by suppressive immune cell accumulation in the tumor microenvironment. Han et al. (2019) found that binding of YTHDF1 to a transcript encoding a lysosomal protease produces an adequate and persistent anti-tumor immune response. Meanwhile, the therapeutic effect of PD-L1 checkpoint blockade was significantly enhanced in YTHDF1 mice.

In a groundbreaking study, Su et al. (2018) found that the oncometabolite R-2-hydroxyglutarate (R-2HG) inhibits FTO activity. In this study, R-2HG was used to directly inhibit FTO in R-2HG-sensitive acute myeloid leukemia and glioma cells, leading to increased methylation and decreased expression of c-MYC and CEBPA mRNAs, thereby enhancing the anti-tumor effects. A study provides evidence of the non-steroidal anti-inflammatory drug Meclofenamic acid (MA) is a highly selective FTO inhibitor, which leads to increased levels of m6A modification in glioblastoma cells, inhibiting tumor progression and prolonging the life span of glioblastoma stem cell (GSC) transplanted mice (Huang et al., 2015; Cui et al., 2017). Recent studies have developed two FTO inhibitors, FB23 and FB23-2, they have been shown to promote differentiation/apoptosis of acute myeloid leukemia cell lines *in vitro*, and significantly inhibit the proliferation of human acute myeloid leukemia (AML) cell lines and primary AML cells, and the progression of human acute myeloid leukemia in xenografted mice (Huang et al., 2019). Deoxycholic acid reduces the expression of miR-92b-3p by m6A-dependent post-transcriptional modification by promoting the dissociation of METTL3 from METTL 3-METTL 14-WTAP complex and plays a role as a tumor suppressor in gallbladder cancer (Lin et al., 2020).

Radiotherapy is a common method for the treatment of tumors. However, many cancer patients develop radiation resistance after radiotherapy. The exact reason is not yet clear. Studies have shown that the silencing METTL3 increases the sensitivity of glioma stem-like cells (Visvanathan et al., 2018), colon cancer cells (Zhang et al., 2019), and pancreatic cancer cells (Taketo et al., 2018) to chemotherapy and radiation resistance. FTO modulates β-catenin expression by decreasing the level of m6A in its gene transcriptome, and increasing the activity of excision repair cross complementation group 1 (ERCC1), thereby enhancing radiotherapy tolerance *in vivo* and *in vitro* (Zhou et al., 2018). In Nasopharyngeal Carcinoma (He et al., 2020), YTHDC2 promotes radiotherapy resistance through activating IGF1R/AKT/S6 Signaling Axis.

Chemotherapy is the standard treatment for a variety of cancers, especially for patients who cannot tolerate surgery; however, acquired chemotherapeutic resistance is one of the major causes of treatment failure. Downregulation of m6A demethylase FTO and ALKBH5 increased the modification of the FZD10mRNA gene to reduce the sensitivity of ovarian epithelial cell carcinoma to PARPI by upregulating the Wnt/β-catenin pathway (Fukumoto et al., 2019). m6A methyltransferase METTL3 mediated autophagy increases the sensitivity of non-small cell lung cancer cells to gefitinib by β-elemene (Liu S. et al., 2020). And METTL3 also directly promotes YAP translation and increases YAP activity by regulating the MALAT1-miR-1914-3p-YAP axis to induce drug resistance and metastasis of non-small cell lung cancer (Jin et al., 2019). m6A-methyladenosine modification modulates the β-catenin signaling pathway to maintain sorafenib resistance in hepatocellular carcinoma (Xu et al., 2020).

According to existing studies, RNA m6 modification not only provides a new idea for male infertility with spermatogenesis disorders but also plays a role in tumorigenesis, showing great potential for early tumor diagnosis, targeted therapy, and improving the sensitivity of chemotherapy drugs and radiotherapy. However, there may still be limitations in the future clinical application of m6A, such as which patients need m6A assistance; Who can afford to pay; Whether the detection of m6A related proteins involves ethics, etc.

## Discussion

As a newly discovered type of post-transcriptional regulation, dynamic reversible m6A modification is the most common type of internal modification for RNA methylation. Although According to previous researches, m6A plays an important role in spermatogenesis, there are still many unsolved problems that need to be further explored, such as the specific mechanism of m6A in spermatogenesis, whether there is a direct link between writer, erasers, and readers. Besides, the majority of the current studies on m6A methylation are focused on animal experiments, which may have specific limitations. What additional external factors can change these enzymes and further affect spermatogenesis. In this review, we found mRNA m6A modifications have been intensely studied in male genital system tumors in the past few years. However, most studies focused on the specific mechanism related to m6A while there is a lack of studies about the application of m6A such as new drug targeting m6A and diagnostic biomarkers based on m6A regulators.

Despite the fruitful results of the preliminary studies, we believe that the following exploratory work still needs to be completed. Above all, more efforts and multi-center, large-scale studies are needed to explore the specific role of m6A in the various pathways that regulate gene expression. Besides, it is still possible that some of the enzymes that modify m6A have not been identified. Such as FMR and HNRNPC may act as a novel m6A binding protein (van Tran et al., 2019). Therefore, the development of new detection methods for m6A will be helpful for the identification of m6A modifiers and the discovery of new regulatory mechanisms. Finally, m6A modifies not only mRNA but also other RNAs including miRNAs (Qi et al., 2019; Thyagarajan et al., 2019), lncRNA (Quinn and Chang, 2016; Yang et al., 2018), and circRNA (Yang et al., 2017; Zhou et al., 2017; Ren C. et al., 2019). Therefore, non-coding RNAs have the potential to become new therapeutic targets for diseases. However, whether there is a potential link between RNA m6A modification and other types of RNA modification remains to be determined.

## Conclusion

According to the reviewed studies, m6A modification plays an important role in spermatogenesis and male genital system tumor occurrence and development. However, the current research is still only at the basic research level. So, to apply in the clinical as soon as possible, we need more efforts and more multicenter, large-scale studies to further explore the role of m6A gene modification in tumor biology. In a word, our research and understanding of the modification of m6A are still in their infancy.

## Author Contributions

SL, YL, and YaW collected the related manuscript and finished the manuscript and figures. ZD gave constructive guidance and made final approval. RL, XF, YuW, and XG participated in the design of this review. All authors read and approved the final manuscript.

## Conflict of Interest

The authors declare that the research was conducted in the absence of any commercial or financial relationships that could be construed as a potential conflict of interest.

## Footnotes

1 https://portal.gdc.com

## References

[B1] Alarcón C. R. Goodarzi H. Lee H. Liu X. Tavazoie S. Tavazoie S. F. (2015). HNRNPA2B1 Is a Mediator of mA-Dependent Nuclear RNA Processing Events. *Cell* 162 1299–1308. 10.1016/j.cell.2015.08.011 26321680 PMC4673968

[B2] Bagrodia A. Lee B. H. Lee W. Cha E. K. Sfakianos J. P. Iyer G. (2016). Genetic Determinants of Cisplatin Resistance in Patients With Advanced Germ Cell Tumors. *J. Clin. Oncol.* 34 4000–4007. 10.1200/jco.2016.68.7798 27646943 PMC5477828

[B3] Bailey A. S. Batista P. J. Gold R. S. Chen Y. G. de Rooij D. G. Chang H. Y. (2017). The conserved RNA helicase YTHDC2 regulates the transition from proliferation to differentiation in the germline. *eLife* 6:e26116. 10.7554/eLife.26116 29087293 PMC5703642

[B4] Bakardjieva-Mihaylova V. Skvarova Kramarzova K. Slamova M. Svaton M. Rejlova K. Zaliova M. (2019). Molecular Basis of Cisplatin Resistance in Testicular Germ Cell Tumors. *Cancers* 11:1316. 10.3390/cancers11091316 31500094 PMC6769617

[B5] Barros-Silva D. Lobo J. Guimarães-Teixeira C. Carneiro I. Oliveira J. Martens-Uzunova E. S. (2020). VIRMA-Dependent N6-Methyladenosine Modifications Regulate the Expression of Long Non-Coding RNAs CCAT1 and CCAT2 in Prostate Cancer. *Cancers* 12:771. 10.3390/cancers12040771 32218194 PMC7226055

[B6] Bell J. L. Wächter K. Mühleck B. Pazaitis N. Köhn M. Lederer M. (2013). Insulin-like growth factor 2 mRNA-binding proteins (IGF2BPs): post-transcriptional drivers of cancer progression? *Cell. Mol. Life Sci.* 70 2657–2675. 10.1007/s00018-012-1186-z 23069990 PMC3708292

[B7] Bokar J. A. Shambaugh M. E. Polayes D. Matera A. G. Rottman F. M. (1997). Purification and cDNA cloning of the AdoMet-binding subunit of the human mRNA (N6-adenosine)-methyltransferase. *RNA* 3 1233–1247. 9409616 PMC1369564

[B8] Bray F. Ferlay J. Soerjomataram I. Siegel R. L. Torre L. A. Jemal A. (2018). Global cancer statistics 2018: GLOBOCAN estimates of incidence and mortality worldwide for 36 cancers in 185 countries. *CA Cancer J. Clin.* 68 394–424. 10.3322/caac.21492 30207593

[B9] Cai J. Yang F. Zhan H. Situ J. Li W. Mao Y. (2019). RNA mA Methyltransferase METTL3 Promotes The Growth Of Prostate Cancer By Regulating Hedgehog Pathway. *Onco Targets Ther.* 12 9143–9152. 10.2147/ott.S226796 31806999 PMC6842310

[B10] Castillo J. J. Mull N. Reagan J. L. Nemr S. Mitri J. (2012). Increased incidence of non-Hodgkin lymphoma, leukemia, and myeloma in patients with diabetes mellitus type 2: a meta-analysis of observational studies. *Blood* 119 4845–4850. 10.1182/blood-2011-06-362830 22496152 PMC3367891

[B11] Chen H. Li Y. Li L. Zhu J. Yang Z. Zhang J. (2020). YTHDC1 gene polymorphisms and hepatoblastoma susceptibility in Chinese children: A seven-center case-control study. *J. Gene Med.* 22:e3249. 10.1002/jgm.3249 32729171

[B12] Chen S. Li Y. Zhi S. Ding Z. Wang W. Peng Y. (2020). WTAP promotes osteosarcoma tumorigenesis by repressing HMBOX1 expression in an mA-dependent manner. *Cell Death Dis.* 11:659. 10.1038/s41419-020-02847-6 32814762 PMC7438489

[B13] Chen Y. Wang J. Xu D. Xiang Z. Ding J. Yang X. (2020). mA mRNA methylation regulates testosterone synthesis through modulating autophagy in Leydig cells. *Autophagy* 17 457–475. 10.1080/15548627.2020.1720431 31983283 PMC8007139

[B14] Chia V. M. Quraishi S. M. Devesa S. S. Purdue M. P. Cook M. B. McGlynn K. A. (2010). International trends in the incidence of testicular cancer, 1973-2002. *Cancer Epidemiol. Biomarkers. Prev.* 19 1151–1159. 10.1158/1055-9965.Epi-10-0031 20447912 PMC2867073

[B15] Chovanec M. Albany C. Mego M. Montironi R. Cimadamore A. Cheng L. (2018). Emerging Prognostic Biomarkers in Testicular Germ Cell Tumors: Looking Beyond Established Practice. *Front. Oncol.* 8:571. 10.3389/fonc.2018.00571 30547014 PMC6280583

[B16] Cui Q. Shi H. Ye P. Li L. Qu Q. Sun G. (2017). mA RNA Methylation Regulates the Self-Renewal and Tumorigenesis of Glioblastoma Stem Cells. *Cell Rep.* 18 2622–2634. 10.1016/j.celrep.2017.02.059 28297667 PMC5479356

[B17] de la Parra C. Ernlund A. Alard A. Ruggles K. Ueberheide B. Schneider R. J. (2018). A widespread alternate form of cap-dependent mRNA translation initiation. *Nat. Commun.* 9:3068. 10.1038/s41467-018-05539-0 30076308 PMC6076257

[B18] Desrosiers R. Friderici K. Rottman F. (1974). Identification of methylated nucleosides in messenger RNA from Novikoff hepatoma cells. *Proc. Natl. Acad. Sci. U.S.A.* 71 3971–3975. 10.1073/pnas.71.10.3971 4372599 PMC434308

[B19] Dina C. Meyre D. Gallina S. Durand E. Körner A. Jacobson P. (2007). Variation in FTO contributes to childhood obesity and severe adult obesity. *Nat. Genet.* 39 724–726. 10.1038/ng2048 17496892

[B20] Dominissini D. Moshitch-Moshkovitz S. Schwartz S. Salmon-Divon M. Ungar L. Osenberg S. (2012). Topology of the human and mouse m6A RNA methylomes revealed by m6A-seq. *Nature* 485 201–206. 10.1038/nature11112 22575960

[B21] Drobnis E. Z. Johnson M. (2015). The question of sperm DNA fragmentation testing in the male infertility work-up: a response to Professor Lewis’ commentary. *Reprod. Biomed. Online* 31 138–139. 10.1016/j.rbmo.2015.05.004 26099439

[B22] Du C. Lv C. Feng Y. Yu S. (2020). Activation of the KDM5A/miRNA-495/YTHDF2/m6A-MOB3B axis facilitates prostate cancer progression. *J. Exp. Clin. Cancer Res.* 39:223. 10.1186/s13046-020-01735-3 33087165 PMC7576758

[B23] Du H. Zhao Y. He J. Zhang Y. Xi H. Liu M. (2016). YTHDF2 destabilizes mA-containing RNA through direct recruitment of the CCR4-NOT deadenylase complex. *Nat. Commun.* 7:12626. 10.1038/ncomms12626 27558897 PMC5007331

[B24] Dubin L. Amelar R. D. (1971). Etiologic factors in 1294 consecutive cases of male infertility. *Fertil. Steril.* 22 469–474. 10.1016/s0015-028238400-x 4398669

[B25] Einhorn L. H. (1993). General Motors Cancer Research Prizewinners Laureates Lectures. Charles F. Kettering Prize. Clinical trials in testicular cancer. *Cancer* 71 3182–3184. 8490849 10.1002/1097-0142(19930515)71:10<3182::aid-cncr2820711046>3.0.co;2-o

[B26] Fanale D. Iovanna J. L. Calvo E. L. Berthezene P. Belleau P. Dagorn J. C. (2014). Germline copy number variation in the YTHDC2 gene: does it have a role in finding a novel potential molecular target involved in pancreatic adenocarcinoma susceptibility? *Expert Opin. Ther. Targets* 18 841–850. 10.1517/14728222.2014.920324 24834797

[B27] Frayling T. M. Timpson N. J. Weedon M. N. Zeggini E. Freathy R. M. Lindgren C. M. (2007). A common variant in the FTO gene is associated with body mass index and predisposes to childhood and adult obesity. *Science* 316 889–894. 10.1126/science.1141634 17434869 PMC2646098

[B28] Fukumoto T. Zhu H. Nacarelli T. Karakashev S. Fatkhutdinov N. Wu S. (2019). N-Methylation of Adenosine of FZD10 mRNA Contributes to PARP Inhibitor Resistance. *Cancer Res.* 79 2812–2820. 10.1158/0008-5472.Can-18-3592 30967398 PMC6548690

[B29] Ghazarian A. A. Kelly S. P. Altekruse S. F. Rosenberg P. S. McGlynn K. A. (2017). Future of testicular germ cell tumor incidence in the United States: Forecast through 2026. *Cancer* 123 2320–2328. 10.1002/cncr.30597 28241106 PMC5629636

[B30] Guzick D. S. Overstreet J. W. Factor-Litvak P. Brazil C. K. Nakajima S. T. Coutifaris C. (2001). Sperm morphology, motility, and concentration in fertile and infertile men. *N. Engl. J. Med.* 345 1388–1393. 10.1056/NEJMoa003005 11794171

[B31] Han D. Liu J. Chen C. Dong L. Liu Y. Chang R. (2019). Anti-tumour immunity controlled through mRNA mA methylation and YTHDF1 in dendritic cells. *Nature* 566 270–274. 10.1038/s41586-019-0916-x 30728504 PMC6522227

[B32] Hanniford D. Ulloa-Morales A. Karz A. Berzoti-Coelho M. G. Moubarak R. S. Sánchez-Sendra B. (2020). Epigenetic Silencing of CDR1as Drives IGF2BP3-Mediated Melanoma Invasion and Metastasis. *Cancer Cell* 37 55–70.e15. 10.1016/j.ccell.2019.12.007 31935372 PMC7184928

[B33] Hartmann A. M. Nayler O. Schwaiger F. W. Obermeier A. Stamm S. (1999). The interaction and colocalization of Sam68 with the splicing-associated factor YT521-B in nuclear dots is regulated by the Src family kinase p59(fyn). *Mol. Biol. Cell* 10 3909–3926. 10.1091/mbc.10.11.3909 10564280 PMC25688

[B34] He J. J. Li Z. Rong Z. X. Gao J. Mu Y. Guan Y. D. (2020). mA Reader YTHDC2 Promotes Radiotherapy Resistance of Nasopharyngeal Carcinoma via Activating IGF1R/AKT/S6 Signaling Axis. *Front. Oncol.* 10:1166. 10.3389/fonc.2020.01166 32850334 PMC7411471

[B35] Hsu P. J. Zhu Y. Ma H. Guo Y. Shi X. Liu Y. (2017). Ythdc2 is an N-methyladenosine binding protein that regulates mammalian spermatogenesis. *Cell Res.* 27 1115–1127. 10.1038/cr.2017.99 28809393 PMC5587856

[B36] Huang H. Weng H. Sun W. Qin X. Shi H. Wu H. (2018). Recognition of RNA N-methyladenosine by IGF2BP proteins enhances mRNA stability and translation. *Nat. Cell Biol.* 20 285–295. 10.1038/s41556-018-0045-z 29476152 PMC5826585

[B37] Huang T. Liu Z. Zheng Y. Feng T. Gao Q. Zeng W. (2020). YTHDF2 promotes spermagonial adhesion through modulating MMPs decay via mA/mRNA pathway. *Cell Death Dis.* 11:37. 10.1038/s41419-020-2235-4 31959747 PMC6971064

[B38] Huang Y. Su R. Sheng Y. Dong L. Dong Z. Xu H. (2019). Small-Molecule Targeting of Oncogenic FTO Demethylase in Acute Myeloid Leukemia. *Cancer Cell* 35 677–691.e10. 10.1016/j.ccell.2019.03.006 30991027 PMC6812656

[B39] Huang Y. Yan J. Li Q. Li J. Gong S. Zhou H. (2015). Meclofenamic acid selectively inhibits FTO demethylation of m6A over ALKBH5. *Nucleic Acids Res.* 43 373–384. 10.1093/nar/gku1276 25452335 PMC4288171

[B40] Huynh T. Mollard R. Trounson A. (2002). Selected genetic factors associated with male infertility. *Hum. Reprod. Update* 8 183–198. 10.1093/humupd/8.2.183 12099633

[B41] Ignatova V. V. Stolz P. Kaiser S. Gustafsson T. H. Lastres P. R. Sanz-Moreno A. (2020). The rRNA mA methyltransferase METTL5 is involved in pluripotency and developmental programs. *Genes Dev.* 34 715–729. 10.1101/gad.333369.119 32217665 PMC7197354

[B42] Iles M. M. Law M. H. Stacey S. N. Han J. Fang S. Pfeiffer R. (2013). A variant in FTO shows association with melanoma risk not due to BMI. *Nat. Genet*. 45 428–432. 10.1038/ng.2571 23455637 PMC3640814

[B43] Jain D. Puno M. R. Meydan C. Lailler N. Mason C. E. Lima C. D. (2018). ketu mutant mice uncover an essential meiotic function for the ancient RNA helicase YTHDC2. *eLife* 7:e30919. 10.7554/eLife.30919 29360036 PMC5832417

[B44] Ji G. Long Y. Zhou Y. Huang C. Gu A. Wang X. (2012). Common variants in mismatch repair genes associated with increased risk of sperm DNA damage and male infertility. *BMC Med.* 10:49. 10.1186/1741-7015-10-49 22594646 PMC3378460

[B45] Jia G. Fu Y. Zhao X. Dai Q. Zheng G. Yang Y. (2011). N6-methyladenosine in nuclear RNA is a major substrate of the obesity-associated FTO. *Nat. Chem. Biol.* 7 885–887. 10.1038/nchembio.687 22002720 PMC3218240

[B46] Jin D. Guo J. Wu Y. Du J. Yang L. Wang X. (2019). mA mRNA methylation initiated by METTL3 directly promotes YAP translation and increases YAP activity by regulating the MALAT1-miR-1914-3p-YAP axis to induce NSCLC drug resistance and metastasis. *J. Hematol. Oncol.* 12:135. 10.1186/s13045-019-0830-6 31818312 PMC6902496

[B47] Kasowitz S. D. Ma J. Anderson S. J. Leu N. A. Xu Y. Gregory B. D. (2018). Nuclear m6A reader YTHDC1 regulates alternative polyadenylation and splicing during mouse oocyte development. *PLoS Genet.* 14:e1007412. 10.1371/journal.pgen.1007412 29799838 PMC5991768

[B48] Kyono K. Fukunaga N. Haigo K. Chiba S. Araki Y. (2001). Pregnancy achieved following ICSI from a man with Klinefelter’s syndrome and spinal cord injury. *Hum. Reprod.* 16 2347–2349. 10.1093/humrep/16.11.2347 11679518

[B49] Landfors M. Nakken S. Fusser M. Dahl J. A. Klungland A. Fedorcsak P. (2016). Sequencing of FTO and ALKBH5 in men undergoing infertility work-up identifies an infertility-associated variant and two missense mutations. *Fertil. Steril*. 105 1170–1179.e5. 10.1016/j.fertnstert.2016.01.002 26820768

[B50] Lee A. S. Kranzusch P. J. Doudna J. A. Cate J. H. (2016). eIF3d is an mRNA cap-binding protein that is required for specialized translation initiation. *Nature* 536 96–99. 10.1038/nature18954 27462815 PMC5003174

[B51] Li E. Wei B. Wang X. Kang R. (2020). METTL3 enhances cell adhesion through stabilizing integrin β1 mRNA via an m6A-HuR-dependent mechanism in prostatic carcinoma. *Am. J. Cancer Res.* 10 1012–1025. 32266107 PMC7136910

[B52] Li F. Zhao D. Wu J. Shi Y. (2014). Structure of the YTH domain of human YTHDF2 in complex with an mA mononucleotide reveals an aromatic cage for mA recognition. *Cell Res.* 24 1490–1492. 10.1038/cr.2014.153 25412658 PMC4260351

[B53] Li J. Xie H. Ying Y. Chen H. Yan H. He L. (2020). YTHDF2 mediates the mRNA degradation of the tumor suppressors to induce AKT phosphorylation in N6-methyladenosine-dependent way in prostate cancer. *Mol. Cancer* 19:152. 10.1186/s12943-020-01267-6 33121495 PMC7599101

[B54] Li M. Zhao X. Wang W. Shi H. Pan Q. Lu Z. (2018). Ythdf2-mediated mA mRNA clearance modulates neural development in mice. *Genome Biol.* 19:69. 10.1186/s13059-018-1436-y 29855337 PMC5984442

[B55] Li N. Kang Y. Wang L. Huff S. Tang R. Hui H. (2020). ALKBH5 regulates anti-PD-1 therapy response by modulating lactate and suppressive immune cell accumulation in tumor microenvironment. *Proc. Natl. Acad. Sci. U.S.A.* 117 20159–20170. 10.1073/pnas.1918986117 32747553 PMC7443867

[B56] Li T. Hu P. S. Zuo Z. Lin J. F. Li X. Wu Q. N. (2019). METTL3 facilitates tumor progression via an mA-IGF2BP2-dependent mechanism in colorectal carcinoma. *Mol. Cancer* 18:112. 10.1186/s12943-019-1038-7 31230592 PMC6589893

[B57] Li Y. Zheng J. N. Wang E. H. Gong C. J. Lan K. F. Ding X. (2020). The m6A reader protein YTHDC2 is a potential biomarker and associated with immune infiltration in head and neck squamous cell carcinoma. *PeerJ* 8:e10385. 10.7717/peerj.10385 33304653 PMC7700739

[B58] Li Z. Qian P. Shao W. Shi H. He X. C. Gogol M. (2018). Suppression of mA reader Ythdf2 promotes hematopoietic stem cell expansion. *Cell Res.* 28 904–917. 10.1038/s41422-018-0072-0 30065315 PMC6123498

[B59] Li Z. Weng H. Su R. Weng X. Zuo Z. Li C. (2017). FTO Plays an Oncogenic Role in Acute Myeloid Leukemia as a N-Methyladenosine RNA Demethylase. *Cancer Cell* 31 127–141. 10.1016/j.ccell.2016.11.017 28017614 PMC5234852

[B60] Lin R. Zhan M. Yang L. Wang H. Shen H. Huang S. (2020). Deoxycholic acid modulates the progression of gallbladder cancer through N-methyladenosine-dependent microRNA maturation. *Oncogene* 39 4983–5000. 10.1038/s41388-020-1349-6 32514152 PMC7314665

[B61] Lin Z. Hsu P. J. Xing X. Fang J. Lu Z. Zou Q. (2017). Mettl3-/Mettl14-mediated mRNA N-methyladenosine modulates murine spermatogenesis. *Cell Res.* 27 1216–1230. 10.1038/cr.2017.117 28914256 PMC5630681

[B62] Liu J. Gao M. Xu S. Chen Y. Wu K. Liu H. (2020). YTHDF2/3 Are Required for Somatic Reprogramming through Different RNA Deadenylation Pathways. *Cell Rep.* 32:108120. 10.1016/j.celrep.2020.108120 32905781

[B63] Liu J. Yue Y. Han D. Wang X. Fu Y. Zhang L. (2014). A METTL3-METTL14 complex mediates mammalian nuclear RNA N6-adenosine methylation. *Nat. Chem. Biol.* 10 93–95. 10.1038/nchembio.1432 24316715 PMC3911877

[B64] Liu S. Li Q. Li G. Zhang Q. Zhuo L. Han X. (2020). The mechanism of mA methyltransferase METTL3-mediated autophagy in reversing gefitinib resistance in NSCLC cells by β-elemene. *Cell Death Dis.* 11:969. 10.1038/s41419-020-03148-8 33177491 PMC7658972

[B65] Lobo J. Costa A. L. Cantante M. Guimarães R. Lopes P. Antunes L. (2019a). mA RNA modification and its writer/reader VIRMA/YTHDF3 in testicular germ cell tumors: a role in seminoma phenotype maintenance. *J. Transl. Med.* 17:79. 10.1186/s12967-019-1837-z 30866959 PMC6416960

[B66] Lobo J. Rodrigues Â. Guimarães R. Cantante M. Lopes P. Maurício J. (2019b). Detailed Characterization of Immune Cell Infiltrate and Expression of Immune Checkpoint Molecules PD-L1/CTLA-4 and MMR Proteins in Testicular Germ Cell Tumors Disclose Novel Disease Biomarkers. *Cancers* 11:1535. 10.3390/cancers11101535 31614500 PMC6826711

[B67] Luxton H. J. Simpson B. S. Mills I. G. Brindle N. R. Ahmed Z. Stavrinides V. (2019). The Oncogene Metadherin Interacts with the Known Splicing Proteins YTHDC1, Sam68 and T-STAR and Plays a Novel Role in Alternative mRNA Splicing. *Cancers* 11:1233. 10.3390/cancers11091233 31450747 PMC6770463

[B68] Ma H. Wang X. Cai J. Dai Q. Natchiar S. K. Lv R. (2019). N(6-)Methyladenosine methyltransferase ZCCHC4 mediates ribosomal RNA methylation. *Nat. Chem. Biol.* 15 88–94. 10.1038/s41589-018-0184-3 30531910 PMC6463480

[B69] Ma L. Chen T. Zhang X. Miao Y. Tian X. Yu K. (2021). The mA reader YTHDC2 inhibits lung adenocarcinoma tumorigenesis by suppressing SLC7A11-dependent antioxidant function. *Redox Biol.* 38:101801. 10.1016/j.redox.2020.101801 33232910 PMC7691619

[B70] Ma X. X. Cao Z. G. Zhao S. L. (2020). m6A methyltransferase METTL3 promotes the progression of prostate cancer via m6A-modified LEF1. *Eur. Rev. Med. Pharmacol. Sci.* 24 3565–3571. 10.26355/eurrev_202004_20817 32329830

[B71] Mao Y. Dong L. Liu X. M. Guo J. Ma H. Shen B. (2019). mA in mRNA coding regions promotes translation via the RNA helicase-containing YTHDC2. *Nat. Commun.* 10:5332. 10.1038/s41467-019-13317-9 31767846 PMC6877647

[B72] Mendel M. Chen K. M. Homolka D. Gos P. Pandey R. R. McCarthy A. A. (2018). Methylation of Structured RNA by the mA Writer METTL16 Is Essential for Mouse Embryonic Development. *Mol. Cell*. 71 986–1000.e11. 10.1016/j.molcel.2018.08.004 30197299 PMC6162343

[B73] Meyer K. D. Patil D. P. Zhou J. Zinoviev A. Skabkin M. A. Elemento O. (2015). 5’ UTR mA Promotes Cap-Independent Translation. *Cell* 163 999–1010. 10.1016/j.cell.2015.10.012 26593424 PMC4695625

[B74] Meyer K. D. Saletore Y. Zumbo P. Elemento O. Mason C. E. Jaffrey S. R. (2012). Comprehensive analysis of mRNA methylation reveals enrichment in 3’ UTRs and near stop codons. *Cell* 149 1635–1646. 10.1016/j.cell.2012.05.003 22608085 PMC3383396

[B75] Mohammad H. P. Barbash O. Creasy C. L. (2019). Targeting epigenetic modifications in cancer therapy: erasing the roadmap to cancer. *Nat. Med.* 25 403–418. 10.1038/s41591-019-0376-8 30842676

[B76] Motzer R. J. Geller N. L. Tan C. C. Herr H. Morse M. Fair W. (1991). Salvage chemotherapy for patients with germ cell tumors. The Memorial Sloan-Kettering Cancer Center experience (1979-1989). *Cancer* 67 1305–1310. 1703917 10.1002/1097-0142(19910301)67:5<1305::aid-cncr2820670506>3.0.co;2-j

[B77] Nettersheim D. Berger D. Jostes S. Kristiansen G. Lochnit G. Schorle H. (2019). N6-Methyladenosine detected in RNA of testicular germ cell tumors is controlled by METTL3, ALKBH5, YTHDC1/F1/F2, and HNRNPC as writers, erasers, and readers. *Andrology* 7 498–506. 10.1111/andr.12612 30903744

[B78] Nilsson J. Skog J. Nordstrand A. Baranov V. Mincheva-Nilsson L. Breakefield X. O. (2009). Prostate cancer-derived urine exosomes: a novel approach to biomarkers for prostate cancer. *Br. J. Cancer* 100 1603–1607. 10.1038/sj.bjc.6605058 19401683 PMC2696767

[B79] Ostermeier G. C. Dix D. J. Miller D. Khatri P. Krawetz S. A. (2002). Spermatozoal RNA profiles of normal fertile men. *Lancet* 360 772–777. 10.1016/s0140-673609899-9 12241836

[B80] Pendleton K. E. Chen B. Liu K. Hunter O. V. Xie Y. Tu B. P. (2017). The U6 snRNA mA Methyltransferase METTL16 Regulates SAM Synthetase Intron Retention. *Cell* 169 824–835.e14. 10.1016/j.cell.2017.05.003 28525753 PMC5502809

[B81] Perry R. P. Kelley D. E. Friderici K. Rottman F. (1975). The methylated constituents of L cell messenger RNA: evidence for an unusual cluster at the 5’ terminus. *Cell* 4 387–394. 10.1016/0092-867490159-2 1168101

[B82] Ping X. L. Sun B. F. Wang L. Xiao W. Yang X. Wang W. J. (2014). Mammalian WTAP is a regulatory subunit of the RNA N6-methyladenosine methyltransferase. *Cell Res.* 24 177–189. 10.1038/cr.2014.3 24407421 PMC3915904

[B83] Pinto R. Vågbø C. B. Jakobsson M. E. Kim Y. Baltissen M. P. O’Donohue M. F. (2020). The human methyltransferase ZCCHC4 catalyses N6-methyladenosine modification of 28S ribosomal RNA. *Nucleic Acids Res.* 48 830–846. 10.1093/nar/gkz1147 31799605 PMC6954407

[B84] Qi L. Gao C. Feng F. Zhang T. Yao Y. Wang X. (2019). MicroRNAs associated with lung squamous cell carcinoma: New prognostic biomarkers and therapeutic targets. *J. Cell. Biochem.* 120 18956–18966. 10.1002/jcb.29216 31241205

[B85] Quinn J. J. Chang H. Y. (2016). Unique features of long non-coding RNA biogenesis and function. *Nat. Rev. Genet.* 17 47–62. 10.1038/nrg.2015.10 26666209

[B86] Ren C. Liu J. Zheng B. Yan P. Sun Y. Yue B. (2019). The circular RNA circ-ITCH acts as a tumour suppressor in osteosarcoma via regulating miR-22. *Artif. Cells Nanomed. Biotechnol.* 47 3359–3367. 10.1080/21691401.2019.1649273 31387405

[B87] Ren W. Lu J. Huang M. Gao L. Li D. Wang G. G. (2019). Structure and regulation of ZCCHC4 in mA-methylation of 28S rRNA. *Nat. Commun.* 10:5042. 10.1038/s41467-019-12923-x 31695039 PMC6834594

[B88] Roundtree I. A. Luo G. Z. Zhang Z. Wang X. Zhou T. Cui Y. (2017). YTHDC1 mediates nuclear export of N-methyladenosine methylated mRNAs. *eLife* 6:e31311. 10.7554/eLife.31311 28984244 PMC5648532

[B89] Scuteri A. Sanna S. Chen W. M. Uda M. Albai G. Strait J. (2007). Genome-wide association scan shows genetic variants in the FTO gene are associated with obesity-related traits. *PLoS Genet.* 3:e115. 10.1371/journal.pgen.0030115 17658951 PMC1934391

[B90] Shi H. Wang X. Lu Z. Zhao B. S. Ma H. Hsu P. J. (2017). YTHDF3 facilitates translation and decay of N-methyladenosine-modified RNA. *Cell Res.* 27 315–328. 10.1038/cr.2017.15 28106072 PMC5339834

[B91] Shi Y. Fan S. Wu M. Zuo Z. Li X. Jiang L. (2019). YTHDF1 links hypoxia adaptation and non-small cell lung cancer progression. *Nat. Commun.* 10:4892. 10.1038/s41467-019-12801-6 31653849 PMC6814821

[B92] Siegel R. L. Miller K. D. Fuchs H. E. Jemal A. (2021). Cancer Statistics, 2021. *CA Cancer J. Clin.* 71 7–33. 10.3322/caac.21654 33433946

[B93] Śledź P. Jinek M. (2016). Structural insights into the molecular mechanism of the mA writer complex. *eLife* 5:e18434. 10.7554/eLife.18434 27627798 PMC5023411

[B94] Söderberg K. C. Kaprio J. Verkasalo P. K. Pukkala E. Koskenvuo M. Lundqvist E. (2009). Overweight, obesity and risk of haematological malignancies: a cohort study of Swedish and Finnish twins. *Eur. J. Cancer* 45 1232–1238. 10.1016/j.ejca.2008.11.004 19091543

[B95] Stoilov P. Rafalska I. Stamm S. (2002). YTH: a new domain in nuclear proteins. *Trends Biochem. Sci.* 27 495–497. 10.1016/s0968-000402189-8 12368078

[B96] Su R. Dong L. Li C. Nachtergaele S. Wunderlich M. Qing Y. (2018). R-2HG Exhibits Anti-tumor Activity by Targeting FTO/mA/MYC/CEBPA Signaling. *Cell* 172 90–105.e23. 10.1016/j.cell.2017.11.031 29249359 PMC5766423

[B97] Sun S. Han Q. Liang M. Zhang Q. Zhang J. Cao J. (2020). Downregulation of m A reader YTHDC2 promotes tumor progression and predicts poor prognosis in non-small cell lung cancer. *Thorac. Cancer* 11 3269–3279. 10.1111/1759-7714.13667 32956555 PMC7606000

[B98] Sun Y. Li S. Yu W. Zhao Z. Gao J. Chen C. (2020). N-methyladenosine-dependent pri-miR-17-92 maturation suppresses PTEN/TMEM127 and promotes sensitivity to everolimus in gastric cancer. *Cell Death Dis.* 11:836. 10.1038/s41419-020-03049-w 33037176 PMC7547657

[B99] Taketo K. Konno M. Asai A. Koseki J. Toratani M. Satoh T. (2018). The epitranscriptome m6A writer METTL3 promotes chemo- and radioresistance in pancreatic cancer cells. *Int. J. Oncol.* 52 621–629. 10.3892/ijo.2017.4219 29345285

[B100] Tanabe A. Tanikawa K. Tsunetomi M. Takai K. Ikeda H. Konno J. (2016). RNA helicase YTHDC2 promotes cancer metastasis via the enhancement of the efficiency by which HIF-1α mRNA is translated. *Cancer Lett.* 376 34–42. 10.1016/j.canlet.2016.02.022 26996300

[B101] Tang C. Klukovich R. Peng H. Wang Z. Yu T. Zhang Y. (2018). ALKBH5-dependent m6A demethylation controls splicing and stability of long 3’-UTR mRNAs in male germ cells. *Proc. Natl. Acad. Sci. U.S.A.* 115 E325–E333. 10.1073/pnas.1717794115 29279410 PMC5777073

[B102] Tang C. Xie Y. Yu T. Liu N. Wang Z. Woolsey R. J. (2020). mA-dependent biogenesis of circular RNAs in male germ cells. *Cell Res.* 30 211–228. 10.1038/s41422-020-0279-8 32047269 PMC7054367

[B103] Thyagarajan A. Tsai K. Y. Sahu R. P. (2019). MicroRNA heterogeneity in melanoma progression. *Semin. Cancer Biol.* 59 208–220. 10.1016/j.semcancer.2019.05.021 31163254 PMC6885122

[B104] Tournaye H. Krausz C. Oates R. D. (2017). Concepts in diagnosis and therapy for male reproductive impairment. *Lancet Diabetes Endocrinol.* 5 554–564. 10.1016/s2213-858730043-2 27395770

[B105] Tsubokura T. Yamazaki H. Masui K. Sasaki N. Shimizu D. Suzuki G. (2018). Comparison of Image-Guided Intensity-Modulated Radiotherapy and Low-dose Rate Brachytherapy with or without External Beam Radiotherapy in Patients with Localized Prostate Cancer. *Sci. Rep.* 8:10538. 10.1038/s41598-018-28730-1 30002393 PMC6043516

[B106] Ueda Y. Ooshio I. Fusamae Y. Kitae K. Kawaguchi M. Jingushi K. (2017). AlkB homolog 3-mediated tRNA demethylation promotes protein synthesis in cancer cells. *Sci. Rep.* 7:42271. 10.1038/srep42271 28205560 PMC5304225

[B107] van Tran N. Ernst F. G. M. Hawley B. R. Zorbas C. Ulryck N. Hackert P. (2019). The human 18S rRNA m6A methyltransferase METTL5 is stabilized by TRMT112. *Nucleic Acids Res.* 47 7719–7733. 10.1093/nar/gkz619 31328227 PMC6735865

[B108] Verza S. Jr. Esteves S. C. (2008). Sperm defect severity rather than sperm Source is associated with lower fertilization rates after intracytoplasmic sperm injection. *Int. Braz. J. Urol.* 34 49–56. 10.1590/s1677-55382008000100008 18341721

[B109] Visvanathan A. Patil V. Arora A. Hegde A. S. Arivazhagan A. Santosh V. (2018). Essential role of METTL3-mediated mA modification in glioma stem-like cells maintenance and radioresistance. *Oncogene* 37 522–533. 10.1038/onc.2017.351 28991227

[B110] Walsh T. J. Grady R. W. Porter M. P. Lin D. W. Weiss N. S. (2006). Incidence of testicular germ cell cancers in U.S. children: SEER program experience 1973 to 2000. Urology 68 402–405. 10.1016/j.urology.2006.02.045 16904461

[B111] Wang L. Hui H. Agrawal K. Kang Y. Li N. Tang R. (2020). m A RNA methyltransferases METTL3/14 regulate immune responses to anti-PD-1 therapy. *EMBO J.* 39:e104514. 10.15252/embj.2020104514 32964498 PMC7560214

[B112] Wang P. Doxtader K. A. Nam Y. (2016). Structural Basis for Cooperative Function of Mettl3 and Mettl14 Methyltransferases. *Mol. Cell.* 63 306–317. 10.1016/j.molcel.2016.05.041 27373337 PMC4958592

[B113] Wang X. Feng J. Xue Y. Guan Z. Zhang D. Liu Z. (2016). Structural basis of N-adenosine methylation by the METTL3-METTL14 complex. *Nature* 534 575–578. 10.1038/nature18298 27281194

[B114] Wang X. Lu Z. Gomez A. Hon G. C. Yue Y. Han D. (2014). N6-methyladenosine-dependent regulation of messenger RNA stability. *Nature* 505 117–120. 10.1038/nature12730 24284625 PMC3877715

[B115] Wang X. Zhao B. S. Roundtree I. A. Lu Z. Han D. Ma H. (2015). N-methyladenosine Modulates Messenger RNA Translation Efficiency. *Cell* 161 1388–1399. 10.1016/j.cell.2015.05.014 26046440 PMC4825696

[B116] Warda A. S. Kretschmer J. Hackert P. Lenz C. Urlaub H. Höbartner C. (2017). Human METTL16 is a N-methyladenosine (mA) methyltransferase that targets pre-mRNAs and various non-coding RNAs. *EMBO Rep.* 18 2004–2014. 10.15252/embr.201744940 29051200 PMC5666602

[B117] Wei J. Yin Y. Zhou J. Chen H. Peng J. Yang J. (2020). METTL3 potentiates resistance to cisplatin through m A modification of TFAP2C in seminoma. *J. Cell Mol. Med.* 24 11366–11380. 10.1111/jcmm.15738 32857912 PMC7576266

[B118] Wen J. Lv R. Ma H. Shen H. He C. Wang J. (2018). Zc3h13 Regulates Nuclear RNA mA Methylation and Mouse Embryonic Stem Cell Self-Renewal. *Mol. Cell.* 69 1028–1038.e6. 10.1016/j.molcel.2018.02.015 29547716 PMC5858226

[B119] Wen S. Wei Y. Zen C. Xiong W. Niu Y. Zhao Y. (2020). Long non-coding RNA NEAT1 promotes bone metastasis of prostate cancer through N6-methyladenosine. *Mol. Cancer* 19:171. 10.1186/s12943-020-01293-4 33308223 PMC7733260

[B120] Whitfield M. Pollet-Villard X. Levy R. Drevet J. R. Saez F. (2015). Posttesticular sperm maturation, infertility, and hypercholesterolemia. *Asian J. Androl.* 17 742–748. 10.4103/1008-682x.155536 26067871 PMC4577583

[B121] Wojtas M. N. Pandey R. R. Mendel M. Homolka D. Sachidanandam R. Pillai R. S. (2017). Regulation of mA Transcripts by the 3′→5′ RNA Helicase YTHDC2 Is Essential for a Successful Meiotic Program in the Mammalian Germline. *Mol. Cell* 68 374–387.e12. 10.1016/j.molcel.2017.09.021 29033321

[B122] Wu Q. Xie X. Huang Y. Meng S. Li Y. Wang H. (2021). N6-methyladenosine RNA methylation regulators contribute to the progression of prostate cancer. *J. Cancer* 12 682–692. 10.7150/jca.46379 33403026 PMC7778550

[B123] Xia H. Zhong C. Wu X. Chen J. Tao B. Xia X. (2018). Mettl3 Mutation Disrupts Gamete Maturation and Reduces Fertility in Zebrafish. *Genetics* 208 729–743. 10.1534/genetics.117.300574 29196300 PMC5788534

[B124] Xiao W. Adhikari S. Dahal U. Chen Y. S. Hao Y. J. Sun B. F. (2016). Nuclear mA Reader YTHDC1 Regulates mRNA Splicing. *Mol. Cell* 61 507–519. 10.1016/j.molcel.2016.01.012 26876937

[B125] Xu J. Wan Z. Tang M. Lin Z. Jiang S. Ji L. (2020). N-methyladenosine-modified CircRNA-SORE sustains sorafenib resistance in hepatocellular carcinoma by regulating β-catenin signaling. *Mol. Cancer* 19:163. 10.1186/s12943-020-01281-8 33222692 PMC7681956

[B126] Xu K. Yang Y. Feng G. H. Sun B. F. Chen J. Q. Li Y. F. (2017). Mettl3-mediated mA regulates spermatogonial differentiation and meiosis initiation. *Cell Res.* 27 1100–1114. 10.1038/cr.2017.100 28809392 PMC5587845

[B127] Yang D. Qiao J. Wang G. Lan Y. Li G. Guo X. (2018). N6-Methyladenosine modification of lincRNA 1281 is critically required for mESC differentiation potential. *Nucleic Acids Res.* 46 3906–3920. 10.1093/nar/gky130 29529255 PMC5934679

[B128] Yang S. Wei J. Cui Y. H. Park G. Shah P. Deng Y. (2019). mA mRNA demethylase FTO regulates melanoma tumorigenicity and response to anti-PD-1 blockade. *Nat. Commun.* 10:2782. 10.1038/s41467-019-10669-0 31239444 PMC6592937

[B129] Yang Y. Fan X. Mao M. Song X. Wu P. Zhang Y. (2017). Extensive translation of circular RNAs driven by N-methyladenosine. *Cell Res.* 27 626–641. 10.1038/cr.2017.31 28281539 PMC5520850

[B130] Yang Y. Huang W. Huang J. T. Shen F. Xiong J. Yuan E. F. (2016). Increased N6-methyladenosine in Human Sperm RNA as a Risk Factor for Asthenozoospermia. *Sci. Rep.* 6:24345. 10.1038/srep24345 27072590 PMC4829835

[B131] Yu R. Li Q. Feng Z. Cai L. Xu Q. (2019). m6A Reader YTHDF2 Regulates LPS-Induced Inflammatory Response. *Int. J. Mol. Sci.* 20:1323. 10.3390/ijms20061323 30875984 PMC6470741

[B132] Yuan Y. Du Y. Wang L. Liu X. (2020). The M6A methyltransferase METTL3 promotes the development and progression of prostate carcinoma via mediating MYC methylation. *J. Cancer* 11 3588–3595. 10.7150/jca.42338 32284755 PMC7150444

[B133] Yue Y. Liu J. Cui X. Cao J. Luo G. Zhang Z. (2018). VIRMA mediates preferential mA mRNA methylation in 3’UTR and near stop codon and associates with alternative polyadenylation. *Cell Discov*. 4:10. 10.1038/s41421-018-0019-0 29507755 PMC5826926

[B134] Zeng M. Dai X. Liang Z. Sun R. Huang S. Luo L. (2020). Critical roles of mRNA mA modification and YTHDC2 expression for meiotic initiation and progression in female germ cells. *Gene* 753:144810. 10.1016/j.gene.2020.144810 32470506

[B135] Zhang X. Wang F. Wang Z. Yang X. Yu H. Si S. (2020). ALKBH5 promotes the proliferation of renal cell carcinoma by regulating AURKB expression in an mA-dependent manner. *Ann. Transl. Med.* 8:646. 10.21037/atm-20-3079 32566583 PMC7290639

[B136] Zhang Y. Kang M. Zhang B. Meng F. Song J. Kaneko H. (2019). mA modification-mediated CBX8 induction regulates stemness and chemosensitivity of colon cancer via upregulation of LGR5. *Mol. Cancer* 18:185. 10.1186/s12943-019-1116-x 31849331 PMC6918584

[B137] Zhang Z. Wang Q. Zhao X. Shao L. Liu G. Zheng X. (2020). YTHDC1 mitigates ischemic stroke by promoting Akt phosphorylation through destabilizing PTEN mRNA. *Cell Death Dis.* 11:977. 10.1038/s41419-020-03186-2 33188203 PMC7666223

[B138] Zhao T. Wang J. Wu Y. Han L. Chen J. Wei Y. (2021). Increased m6A modification of RNA methylation related to the inhibition of demethylase FTO contributes to MEHP-induced Leydig cell injury(*star*). *Environ. Pollut.* 268 (Pt A):115627. 10.1016/j.envpol.2020.115627 33010548

[B139] Zhao T. X. Wang J. K. Shen L. J. Long C. L. Liu B. Wei Y. (2020). Increased m6A RNA modification is related to the inhibition of the Nrf2-mediated antioxidant response in di-(2-ethylhexyl) phthalate-induced prepubertal testicular injury. *Environ. Pollut.* 259:113911. 10.1016/j.envpol.2020.113911 31923814

[B140] Zheng G. Dahl J. A. Niu Y. Fedorcsak P. Huang C. M. Li C. J. (2013). ALKBH5 is a mammalian RNA demethylase that impacts RNA metabolism and mouse fertility. *Mol. Cell* 49 18–29. 10.1016/j.molcel.2012.10.015 23177736 PMC3646334

[B141] Zhou C. Molinie B. Daneshvar K. Pondick J. V. Wang J. Van Wittenberghe N. (2017). Genome-Wide Maps of m6A circRNAs Identify Widespread and Cell-Type-Specific Methylation Patterns that Are Distinct from mRNAs. *Cell Rep.* 20 2262–2276. 10.1016/j.celrep.2017.08.027 28854373 PMC5705222

[B142] Zhou S. Bai Z. L. Xia D. Zhao Z. J. Zhao R. Wang Y. Y. (2018). FTO regulates the chemo-radiotherapy resistance of cervical squamous cell carcinoma (CSCC) by targeting β-catenin through mRNA demethylation. *Mol. Carcinog.* 57 590–597. 10.1002/mc.22782 29315835

[B143] Zhu K. Li Y. Xu Y. (2021). The FTO mA demethylase inhibits the invasion and migration of prostate cancer cells by regulating total mA levels. *Life Sci.* 271:119180. 10.1016/j.lfs.2021.119180 33571513

[B144] Zhu T. Roundtree I. A. Wang P. Wang X. Wang L. Sun C. (2014). Crystal structure of the YTH domain of YTHDF2 reveals mechanism for recognition of N6-methyladenosine. *Cell Res.* 24 1493–1496. 10.1038/cr.2014.152 25412661 PMC4260350

